# DNA methylation for cervical cancer screening: a training set in China

**DOI:** 10.1186/s13148-020-00885-7

**Published:** 2020-06-23

**Authors:** Linghua Kong, Linhai Wang, Ziyun Wang, Xiaoping Xiao, Yan You, Huanwen Wu, Ming Wu, Pei Liu, Lei Li

**Affiliations:** 1 Department of Obstetrics and Gynecology, Peking Union Medical College Hospital, Shuaifuyuan No. 1, Dongcheng District, Beijing, 100730 China; 2 Beijing SinoMDgene Technology Co., Ltd., Floor 3, Building14, Guo Sheng Science Park, No. 1 Kangding Street, Beijing Economic and Technological Development District, Beijing, 100176 China; 3 Department of Pathology, Peking Union Medical College Hospital, Beijing, 100730 China

**Keywords:** Cervical cancer, Cervical intraepithelial neoplasia, DNA methylation, High-risk human papillomavirus, Cytology, Training set

## Abstract

**Background:**

Despite rapid improvements in DNA methylation tools for cervical cancer screening, few robust, exploratory studies have been performed using the combination of two host genes, *EPB41L3* and *JAM3*, newly developed assays.

**Methods:**

A review of abnormal liquid-based cytology and/or high-risk human papillomavirus (hrHPV) data from outpatient clinics in the study center from March 2018 to March 2019 was performed. Eligible patients with definitive histological pathology results were included, and their residual cytology samples were assessed for *EPB41L3* and *JAM3* methylation. The diagnostic accuracies of various screening strategies for definitive pathology and for cervical intraepithelial neoplasia (CIN) 2 or more severe lesions (CIN2+) were compared.

**Results:**

In total, 306 patients were successfully tested; 301 cases with cervical histological pathology were included in the final analysis, including 118 (39.2%) and 183 (60.8%) cases of inflammation/CIN1 and CIN2+, respectively. Regarding CIN2+ detection, methylation status and hrHPV plus methylation had similar positive predictive values (0.930 and 0.954, respectively, *p* = 0.395). Additionally, hrHPV, methylation, and hrHPV plus methylation had similar negative predictive values (0.612, 0.679, and 0.655, *p* = 0.677) that were significantly higher than that of cytology alone (0.250, *p* values 0.012, 0.001, and 0.001, respectively). For 49 cases with negative hrHPV results, positive methylation alone was able to differentiate CIN2+ from inflammation/CIN1.

**Conclusions:**

Methylation of both *EPB41L3* and *JAM3* is an accurate and feasible screening method for CIN2+.

## Introduction

Uterine cervical cancer is one of most common causes of cancer-related death among women worldwide [1]. According to a conservative estimate, the total incidence and mortality of cervical cancer in China were 98,900 and 30,500 cases, respectively, in 2015 [2], accounting for one-fifth of the total number of new cases of cervical cancer worldwide [3]. However, great discrepancies exist in the popularity and quality of screening methods [4]. Although a combination of cervical cytology and/or high-risk human papillomavirus (hrHPV) in women of relevant age is the mainstream screening method [5, 6], cytology has several limitations, including a lack of high-throughput characteristics, requirement for high-level skills by pathologists, and low sensitivity [7, 8]. The limitations of hrHPV testing pertain to its high cost, low specificity, and possible reproducibility difficulties given the large Chinese population and territory. Inevitably, the cost versus benefit is key for the decision to undergo cervical cancer screening, even though defining an acceptable risk will likely differ between settings [9]. Thus, a new screening strategy with high accuracy and feasibility is urgently needed.

Increasing evidence has shown that epigenetic silencing of tumor-suppressor genes is essential to carcinogenesis and metastasis [10]. The actions of one epigenetic mechanism, DNA methylation, result in the heritable silencing of genes without changes in their coding sequences [11], thereby affecting virtually every step in tumor progression [12]. DNA methylation is also essential for the progression and pathogenesis of cervical cancer, as reflected in its sensitivity for prognosis and therapy in clinical practice. As genotyping and methylation markers are objective and applicable to self-collected samples, these approaches offer logistical advantages, including accessibility in low- and middle-income settings [9]. More than 100 human (host) genes have been reported to be possible methylation biomarkers of cervical cancer [13]. Furthermore, numerous studies have shown that methylation has high screening sensitivity for lesions of cervical intraepithelial neoplasia (CIN) 2 or more severe lesions (CIN2+ or high-grade intraepithelial lesions [HSIL]) and can be used as a triage method in women with positive hrHPV status. Multiple panels consisting of dozens of candidate host genes, virus genes or both, as well as various combinations, have been utilized as classifiers [14]. A number of studies have explored the role of a panel including *EPB41L3* [15–24], *JAM3* [25, 26], or both [7, 27–31] in the screening or triage of HSIL and/or cervical cancer.

In this exploratory study, we selected a cohort with definitive cervical biopsy pathology findings after abnormal cytology and/or hrHPV testing so as to develop an assay of *EPB41L3* and *JAM3* methylation. Residual liquid-based cytology samples were assessed for *EPB41L3* and *JAM3* methylation, and *EPB41L3* and *JAM3* methylation status was determined for discriminating CIN2+ from normal cervical findings/CIN1 (or low-grade intraepithelial lesions [LSIL]) according to known cervical histological pathology. The diagnostic accuracy of DNA methylation was compared with that of hrHPV-based strategies.

## Methods

### Ethical approval

The Institutional Review Board from the study center approved the study (No. JS-1954). All patients provided their consent before enrollment. The registration number is NCT03961191 (*clinicaltrials.gov*, registered on May 23, 2019). All procedures performed in the study involving human participants were in accordance with the ethical standards of the institutional and National Research Committee and with the 1964 *Declaration of Helsinki* and its later amendments or comparable ethical standards.

### Study design

This study is to develop an assay of *EPB41L3* and *JAM3* methylation for detection of CIN2+. A review of data regarding abnormal cytology and/or hrHPV testing results for patients who visited outpatient clinics from March 2018 to March 2019 was performed. We attempted to find 200 cases of inflammation or CIN1, 200 cases of CIN2/3, and 200 cases of cervical carcinomas diagnosed by histological pathology. After registration of the study in May 2019, the eligible patients were asked to return to the outpatient clinics to sign consent forms to participate in the study, and their residual cytology samples were sent for DNA methylation analysis and hrHPV genotyping. The primary endpoints were the cutoff values of *EPB41L3* and *JAM3* methylation and their diagnostic accuracies regardless of hrHPV status in liquid-based cytology specimens for CIN2+*.* The secondary endpoint was comparison of the diagnostic accuracies of various screening methods. However, this comparison would be confirmed in future validation set due to the great bias in this training set caused by enrollment of patients.

### Patient enrollment and sample size

We planned to perform DNA methylation analysis of 300 cases, comprising three groups of 100 cases of inflammation/CIN1, CIN2/3 (HSIL), or cervical carcinoma. After considering factors including patient unwillingness to participate in the study and insufficient residual cytology samples for analysis, we planned to collect information on abnormal cytology and/or hrHPV for 600 cases from patient records. Data for epidemiological characteristics and medical history were obtained from medical records and supplemented by patient interviews. The inclusion criteria consisted of the following: aged 18 or older; abnormal cytology and/or hrHPV according to the criteria of the American Cancer Society, American Society for Colposcopy and Cervical Pathology, and American Society for Clinical Pathology screening guidelines [5] and its updated version [6] for further intervention; available residual cytology samples for methylation analysis in the study center; definitive cervical histological pathology findings in the study center; no history of precancerous cervical lesions or other cancers; no history of medical radiotherapy; negative HIV results and no history of organ transplantation or immunosuppressive therapy; and willingness to participate in the study. Cases not meeting all the criteria were excluded. In addition, to avoid bias, less common histological subtypes of cervical adenocarcinoma (ADC) were also excluded except for endocervical ADC or usual-type ADC, which is the most common ADC type and is regarded as HPV-associated tumor [32]. Patients with endometrial and ovarian cancer underwent methylation testing but were not included in the final analysis for cutoff values. All histological materials were re-evaluated by two pathologists (YY and HW).

### Collection of study materials

The residual cytology samples were obtained from outpatient clinics of the Department of Obstetrics and Gynecology of the study center. These samples had previously been tested for cytology and/or hrHPV. All the samples were stored in PreservCyt Solution (Thinprep Pap Test; Hologic, USA) at room temperature. The cytology evaluation was performed using a Thinprep 2000 (Hologic, USA), and the results are reported according to the Bethesda 2014 system [33]. Primary hrHPV analysis utilized the Cobas 4800 System (Roche Molecular Systems, Inc., USA, only for HPV 16, 18, and others subtypes not specified). However, because primary hrHPV testing results were not available for all patients, the samples were subjected to hrHPV genotyping analysis as well as methylation analysis.

A 2-ml residual cytology sample was collected. Genomic DNA was extracted with the TIANamp Genomic DNA kit (Tiangen Biotech Co., Ltd, China), and the concentration was measured with a Nanodrop-300 microspectrophotometer (Thermo Fisher Scientific Inc., USA). Modification of the isolated DNA was performed using EZ-96 DNA Methylation-Lighting^TM^ MagPrep (Zymo Research CO., USA) according to the manufacturer’s instructions. The standard conversion amount of genomic DNA was 1 μg. The DNA yield was less than 1 μg (23% of the total), and a minimal input of 70 ng was used. The bisulfite-converted DNA was eluted with 40 μL elution buffer and used as the template for polymerase chain reaction (PCR).

### DNA methylation testing

Methylation of the *EPB41L3* and *JAM3* genes was evaluated using TaqMan-based technologies with the Methylated Human *EPB41L3* and *JAM3* Gene Detection kit (real-time fluorescent PCR) (Beijing SinoMDgene Technology Co., LTD, China) and an ABI 7300 Real Time Fluorescence Quantitative PCR system (Life Tech, USA). According to the manufacturer’s recommendations, PCRs were performed in a total volume of 25 μl, containing 15 μl of methylation-specific PCR mix, 5 μl of bisulfite-converted DNA, and optimized concentrations of primers and probes. Leukocyte DNA and HeLa DNA treated with sodium bisulfite were used as negative and positive controls, respectively. Moreover, a nontemplate control was tested in each run to monitor the PCR specificity. The PCR conditions were as follows: 96 °C for 10 min, followed by 45 cycles at 94 °C for 15 s, 64 °C for 5 s, and 60 °C for 30 s.

Three sets of primers and probes were designed using Primer Premier 5: one set for *GAPDH*, which does not contain CpGs, with both methylated and unmethylated sequences amplified equally, and was used as an internal reference to evaluate total bisulfite conversion; two sets for the candidate genes *EPB41L3* and *JAM3*, specifically amplifying the methylated locus of interest. For each sample, the methylation level of each gene was determined by the ΔCt value (ΔCtE = Ct_EPB41L3_ − Ct_GAPDH_; ΔCtJ = Ct_JAM3_ − Ct_GAPDH_). Raw results were exported from the system, and the ΔCt value was calculated. If no amplification for *EPB41L3* or *JAM3* occurred, the Ct value was regarded as 45. When the ΔCt value for at least one of the targets was below its cutoff which was determined as described later, the sample was considered “positive”; when both genes were above their cutoffs, the sample was considered “negative.” A sample was regarded as “invalid” when the GAPDH Ct value was above its cutoff (≥ 35.75). The expression of DNA methylation in any of both genes is defined “positive.”

### Genotyping of hrHPV

hrHPV genotyping was performed with TaqMan-based technology using an ABI 7500 Real Time Fluorescence Quantitative PCR system (Life Tech, USA) or a Stratagene Mx3000p Fluorescence Quantitative PCR system (Stratagene, USA) with an HPV nucleic acid genotyping diagnostic kit (Real time Fluorescent PCR) (Beijing SinoMDgene Technology Co., Ltd, China). The diagnostic kit, a quantitative in vitro assay, detects a pooled result for hrHPV types, including HPV 16, 18, 31, 33, 45, 52, 6, 11, 35, 51, 39, 59, 68, 56, 58, and 66, with type-specific probes. Briefly, 10 ng DNA was added per well, denatured, and combined with a type-specific probes derived from high-risk HPVs. PCR was performed as follows: (1) reaction with the UDG enzyme at 37 °C for 2 min; (2) initial denaturation at 95 °C for 3 min; and (3) denaturation at 94 °C for 15 s and annealing at 60 °C for 45 s, for a total of 40 cycles. Her2, labeled with CY5 channel, was used as internal control and added per well. Results showing the Ct values over 36 for Her2 were defined as detection failures. Samples with a Ct value in FAM channel (for HPV6, 16, 31, 35, 39, 45, 58, and 68, respectively) or HEX channel (for HPV 11, 18, 33, 51, 52, 56, 59, and 66, respectively) no more than 36 were recorded as positive. A positive hrHPV plus methylation status indicates hrHPV positivity as well as methylation positivity for *EPB41L3* or *JAM3* (or both).

### Statistics

The cutoff values of DNA methylation were calculated with a receiver operating characteristic curve (ROC) and Youden index analysis (specificity + sensitivity − 1) based on comparison between cervical inflammation/CIN1 and CIN2 or more severe cervical lesions. Nonnormally distributed variables and categorical data were compared between different screening groups by using nonparametric tests. The specificity, sensitivity, negative predictive value (NPV), and positive predictive value (PPV) in various screening groups were also calculated. The odds ratio (OR) and 95% confidence interval (95% CI) of positive ratios of different screening methods for various histological types were calculated with logistic regression models. Unless otherwise stated, all analyses were performed with a two-sided significance level of 0.05 and were conducted with the use of the Statistical Product and Service Solutions (SPSS) Statistics 20.0 software (IBM Corporation, Armonk, USA).

## Results

### Patient characteristics

**Fig. 1:**
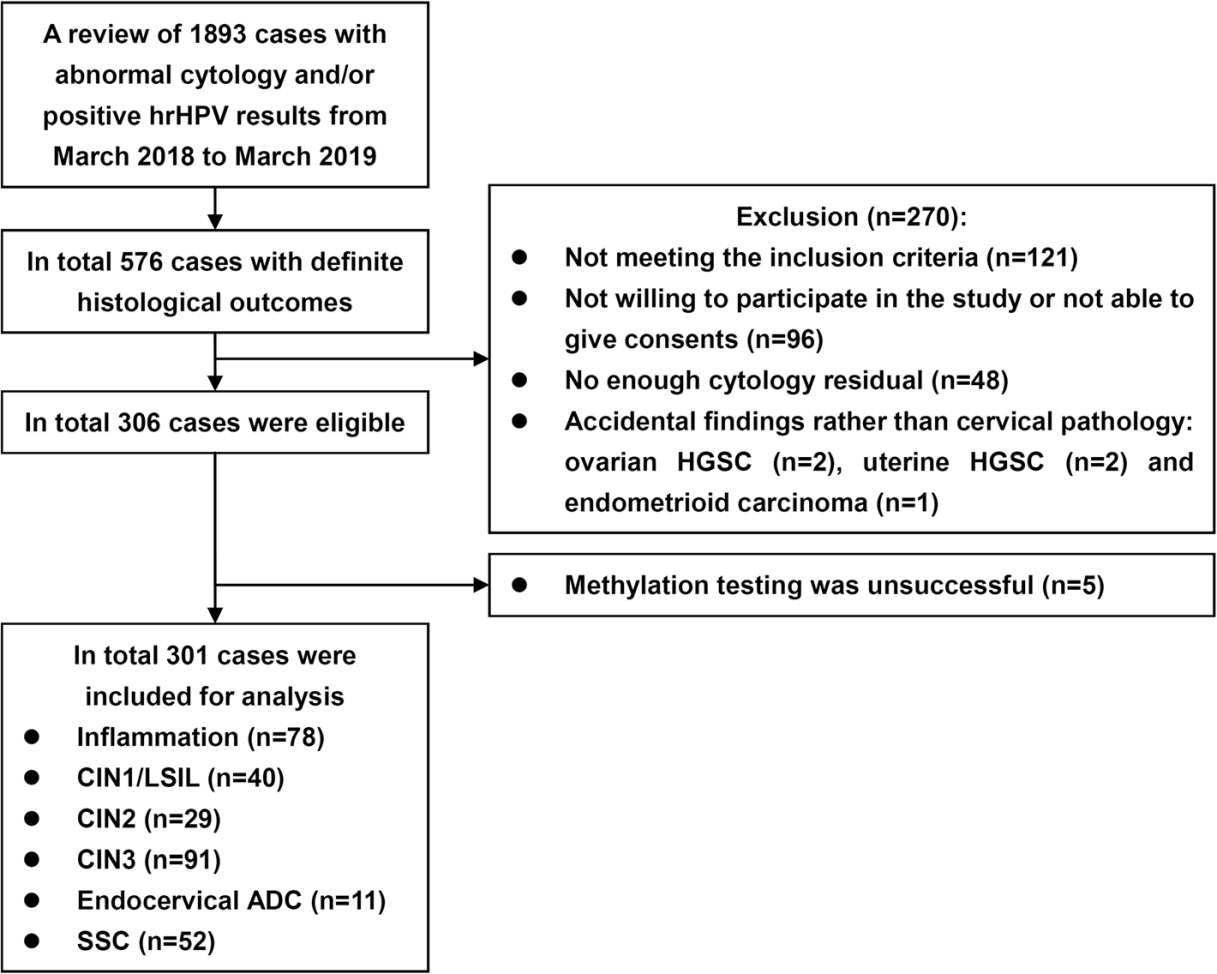
Flow diagram of the study. ADC, adenocarcinoma. CIN, cervical intraepithelial neoplasia. HGSC, high-grade serous carcinoma. hrHPV, high-risk human papilloma virus. SCC, squamous cell carcinoma

A flow diagram of the study is presented in Fig. 1. After reviewing the records of 1893 cases with available cytology data, 576 had definitive histological outcomes, and 306 were eligible; 301 patients accepted methylation testing and included in the final analysis for the cutoff values of DNA methylation. The cohort comprised 78 cases (25.91%) of cervical inflammation, 40 (13.29%) of CIN1, 29 (9.63%) of CIN2, 91 (30.23%) of CIN3, 52 (17.28%) of squamous cell carcinoma (SCC), and 11 (3.65%) of endocervical ADC. These patients represented 52.68% (118/224) cases of inflammation or CIN1, 55.77% (29/52) of CIN2, 50.56% (91/180) of CIN3, and 59.09% (52/88) and 34.38% (11/32) of ADC in discovered 576 cases with definitive histological outcomes. Most (295 cases, 98.01%) were diagnosed by colposcopy with biopsy or by biopsy alone, and six cases (1.99%) were diagnosed with LEEP. Another five cases with abnormal cytology results had been proven to have other gynecologic malignancies rather than cervical lesions.

For all 301 patients, the median age was 45 years (range 25–77), and most (208 of 301, 69.10%) were premenopausal. In total, 32 (10.63%) of 301 patients, 49 (16.28%) of 301 patients, and 37 of 232 patients (15.95%) had negative cytology results, genotyping hrHPV results, and Roche genotyping hrHPV results, respectively (Supplementary Table 1). Similar average age was found between patients with positive and negative hrHPV results (47.88 ± 13.48 vs 44.98 ± 11.58, *p* = 0.120), patients with normal cytology results or ASCUS+ (42.28 ± 9.98 vs 45.83 ± 12.10, *p* = 0.112), and patients with inflammation or CIN1 and CIN2+ (44.00 ± 11.98 vs 46.39 ± 11.84, *p* = 0.090).

### Cutoff values of DNA methylation

**Fig. 2:**
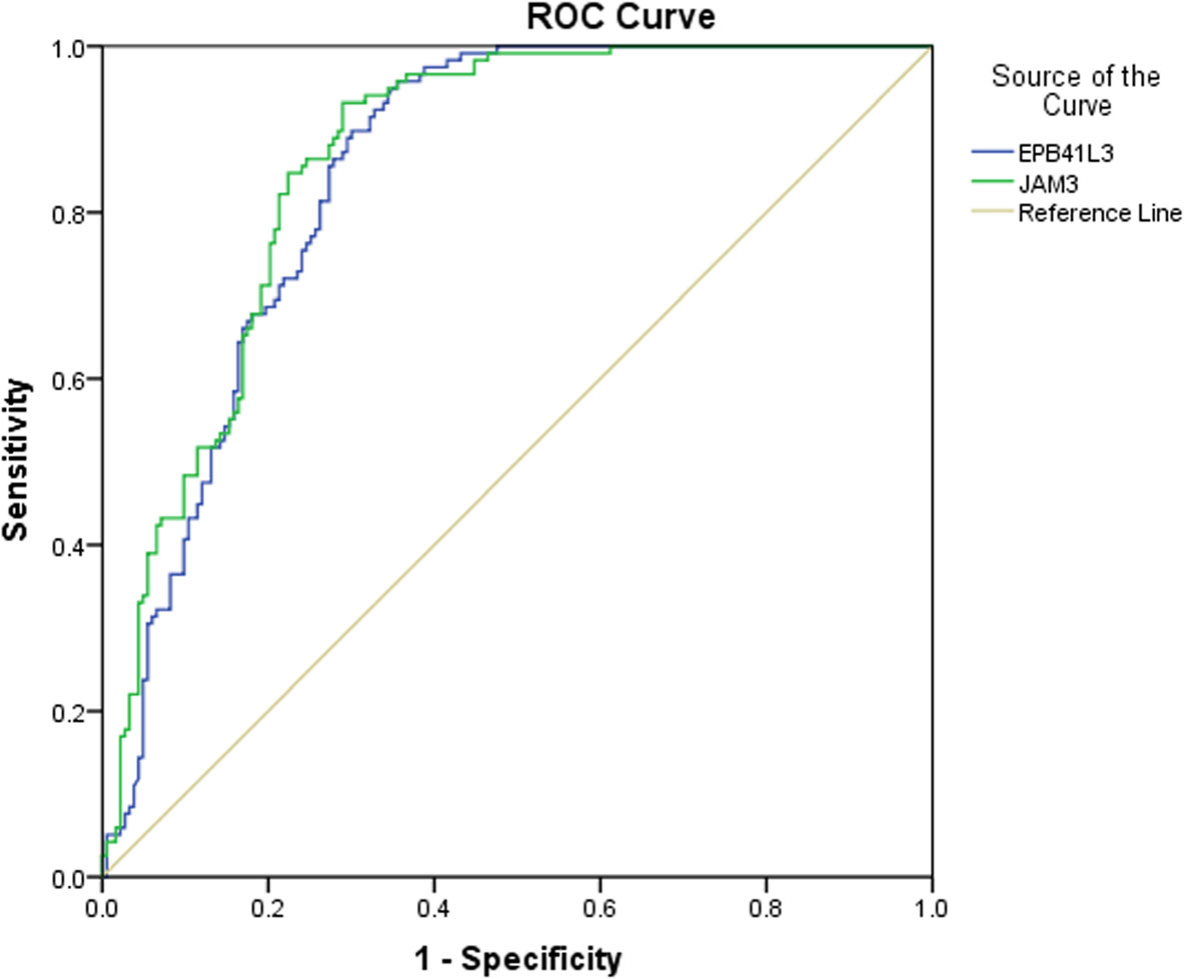
The ROC curve of *EPB41L3* and *JAM3* methylation to differentiate cervical inflammation/cervical intraepithelial neoplasia (CIN) 1 from CIN2 or more severe cervical lesions. For *EPB41L3* and *JAM3*, the areas under the ROC curve were 0.846 and 0.863 (95% confidence interval 0.803 to 0.889 [*p* < 0.001] and 0.822 to 0.903 [*p* < 0.001]), respectively

DNA methylation testing failed in 5 of 311 cases (Fig. 1), all due to the breakdown of cells in the residual liquid-based cytology samples. The specific methylation results are listed in Supplementary Table 1. Based on these figures, the ROC curve for *EPB41L3* and *JAM3* methylation in differentiating cervical inflammation/CIN1 from CIN2+ is shown in Fig. 2. Methylation of both *EPB41L3* and *JAM3* showed high areas under the curve (0.846 and 0.863 (95% CI 0.803 to 0.889 [*p* < 0.001] and 0.822 to 0.903 [*p* < 0.001], respectively). Based on sensitivity, specificity, and Youden index analyses, the cutoff values for *EPB41L3* and *JAM3* methylation were 7.945 and 9.250, respectively, with the highest Youden index values being 0.603 and 0.642, respectively. There were 123 (40.86%), 138 (45.85%), and 142 (47.18%) cases positive for methylation of *EPB41L3*, *JAM3*, and either of the two genes, respectively (Table 1). Patients with positive results for *EPB41L3* (49.20 ± 11.07 vs 42.87 ± 11.84, *p* < 0.001) and *JAM3* (48.49 ± 11.50 vs 42.88 ± 11.71, *p* < 0.001) methylation or for total methylation status (48.51 ± 11.40 vs 42.72 ± 11.76, *p* < 0.001) were significantly older than patients with negative results.

**Table 1:** The association between histological findings and methylation analysis with and without hrHPV testing

Histology		Positive^a^*EPB41L3*, *n* (%)	Positive^a^*JAM3*, *n* (%)	Positive *EPB41L3* or positive *JAM3*, *n* (%)	Positive hrHPV, *n* (%)	Positive hrHPV + methylation, *n* (%)
Inflammation or LSIL (*n* = 118)	Inflammation (*n* = 78)	4 (5.13)	7 (8.97)	8 (10.26)	55 (70.51)	5 (9.09)
	CIN1 (*n* = 40)	1 (2.50)	1 (2.50)	2 (5.00)	31 (77.50)	1 (3.23)
HSIL (*n* = 120)	CIN2 (*n* = 29)	3 (10.34)	5 (17.24)	6 (20.69)	21 (72.41)	4 (19.05)
	CIN3 (*n* = 91)	56 (61.54)	64 (70.33)	65 (71.43)	83 (91.21)	61 (73.49)
Malignancies (*n* = 68)	SCC (*n* = 52)	51 (98.08)	51 (98.08)	51 (98.08)	49 (94.23)	49 (94.23)
	Endocervical ADC (*n* = 11)	8 (72.73)	10 (90.91)	10 (90.91)	9 (81.82)	9 (81.82)

*ADC* adenocarcinoma, *CIN* cervical intraepithelial neoplasia, *HGSC* high-grade serous carcinoma, *hrHPV* high-risk human papilloma virus, *SSC* squamous cell carcinoma

^a^Positive methylation status indicates calculated values below the cutoff values

### Diagnostic accuracies of various screening methods

The ORs for the positive ratios of different screening methods for various histological types are listed in Table 2. With inflammation as the reference, significantly higher ratios for a positive methylation status were found for CIN3 (OR 21.875, 95% CI 9.244–51.764, *p* < 0.001), SCC (446.250, 54.106–3680.553, *p* < 0.001), endocervical ADC (87.500, 9.872–775.520, *p* < 0.001), and other gynecological malignancies (35.000, 3.474–352.665, *p* = 0.003). Although hrHPV positivity showed significantly higher ratios in CIN3 and SCC, hrHPV positivity plus methylation status had significantly higher ratios in CIN3, SCC, and endocervical ADC (all *p* values < 0.05). With inflammation or CIN1 as the reference, significantly higher ratios were observed for positive methylation status, hrHPV status, and hrHPV plus methylation status in CIN2+ (ORs = 27.953, 2.943, and 39.232, all *p* values < 0.001).

**Table 2:** The odds ratio (OR) and its 95% confidence interval (95% CI) of positive ratios of different screening methods in various histological types

	OR (95% CI) of positive methylation^a^	*p*	OR (95% CI) of positive hrHPV^a^	*p*	OR (95% CI) of positive methylation and hrHPV^a^	*p*
Inflammation (*n* = 78)	Reference	–	Reference	–	Reference	–
CIN1 (*n* = 40)	0.461 (0.093–2.279)	0.342	1.269 (0.519–3.106)	0.602	0.374 (0.042–3.318)	0.377
CIN2 (*n* = 29)	2.283 (0.717–7.271)	0.163	1.158 (0.432–3.106)	0.771	3.042 (0.810–11.416)	0.099
CIN3 (*n* = 91)	21.875 (9.244–51.764)	**< 0.001**	4.421 (1.763–11.804)	**0.002**	29.687 (10.856–81.178)	**< 0.001**
SCC (*n* = 52)	446.250 (54.106–3680.553)	**< 0.001**	6.018 (1.692–21.396)	**0.006**	238.467 (54.479–1043.821)	**< 0.001**
Endocervical ADC (*n* = 11)	87.500 (9.872–775.520)	**< 0.001**	1.658 (0.331–8.309)	0.539	65.700 (11.079–389.600)	**< 0.001**
Inflammation or CIN1 (*n* = 118)	Reference	–	Reference	–	Reference	–
CIN2+ (*n* = 183)	27.953 (13.552–57.656)	**< 0.001**	2.943 (1.567–5.527)	**0.001**	39.232 (16.308–94.381)	**< 0.001**

*ADC* adenocarcinoma, *CIN* cervical intraepithelial neoplasia, *CIN2+* lesions of CIN2 or more severe, *HGSC* high grade serous carcinoma, *hrHPV* high risk human papillomavirus, *N/A* not available, *SSC* squamous cell carcinoma

^a^Positive methylation status indicates calculated values of at least one of the targeted genes below the cutoff values

The diagnostic accuracies for CIN2+ are listed in Table 3. As the entire study cohort had abnormal cytology and/or hrHPV testing results, comparisons of sensitivity and specificity were limited to methylation status and methylation plus hrHPV status. These two screening methods exhibited a similar sensitivity (72.13% and 67.76%, *p* = 0.362) and specificity (91.53% and 94.92%, *p* = 0.300). Conversely, cytology alone and hrHPV displayed very poor specificities of 3.39% and 25.42%, respectively.

**Table 3:** The diagnostic accuracy of different screening strategies for cervical histology

Histology of cervix	Cytology		hrHPV		*EPB41L3* or *JAM3*		hrHPV+ methylation	
	Negative	ASCUS+	Negative	Positive	Negative	Positive^a^	Negative	Positive^a^
Inflammation or CIN1 (*n* = 118)	4	114	30	88	108	10	112	6
CIN2+ (*n* = 183)	12	171	19	164	51	132	59	124
Sensitivity for CIN2+	93.44%		89.62%		72.13%		67.76%	
Specificity for CIN2+	3.39%		25.42%		91.53%		94.92%	
Negative predictive value for CIN2+	0.250		0.612		0.679		0.655	
Positive predictive value for CIN2+	0.600		0.651		0.930		0.954	

*ASCUS* atypical squamous cells of undetermined significance, *CIN2+* lesions of cervical intraepithelial neoplasia (CIN) 2 or more severe, *hrHPV* high-risk human papilloma virus, *N/A* not available

^a^Positive methylation status indicates calculated values of at least one of the targeted genes below the cutoff values

In detecting CIN2+, cytology alone, hrHPV alone, and cytology plus hrHPV showed similar PPVs (0.600, 0.651, and 0.608, respectively, *p* = 0.122). However, methylation status and hrHPV plus methylation had similar PPVs (0.930 and 0.954, respectively, *p* = 0.395).

To detect CIN2+, similar NPVs were calculated for hrHPV, methylation, and hrHPV plus methylation (0.612, 0.679, and 0.655, *p* = 0.677), which were all significantly higher than that of cytology alone (0.250, *p* values were 0.012, 0.001, and 0.001, respectively).

In 49 cases with negative hrHPV results, positive methylation alone was able to differentiate CIN2+ from inflammation/CIN1 (8/19 [42.10%] versus 4/30 [13.33%], OR 4.727, 95% CI 1.175–19.016, *p* = 0.027). The sensitivity, specificity, PPV, and NPV of methylation were 42.10%, 86.67%, 0.678, and 0.703, respectively, among cases with negative hrHPV results.

For all patients undergoing methylation testing, the positive ratios of *EPB41L3* and *JAM3* were not significantly different (123/301 [40.9%] versus 138/301 [45.8%], *p* = 0.217). These two genes had similar sensitivities, specificities, NPVs, and PPVs for diagnosing CIN2+ (all *p* values > 0.05).

## Discussion

In this cohort, we defined cutoff values for *EPB41L3* and *JAM3* to differentiate CIN2+ from other cervical lesions. The selection of specific DNA was based on previous studies, which revealed the good performance of *EPB41L3* [20, 34] and *JAM3* [25]. The targeted CpG sites of methylation from *EPB41L3* and *JAM3* genes locate in the promoter and exon 1, respectively. DNA methylation of these two genes showed areas under the ROC curve of 0.846 and 0.863, respectively, which were similar to or better than previous reports of *EPB41L3* and *JAM3* and other genes for the discrimination of HSIL+ from < HSIL cytology [19, 35, 36]. Moreover, the combined methylation status had a good sensitivity, specificity, PPV, and NPV. Because we selected a population with abnormal cytology and/or hrHPV findings, analysis of the diagnostic accuracy of hrHPV and/or cytology would not reflect the real-world situation. Nonetheless, methylation of *EPB41L3* and *JAM3* with or without hrHPV testing achieved the best specificities and PPVs and similar NPVs to those of hrHPV status. It has been reported that there is no association between the methylation of any gene and the presence of human papillomavirus [35]. In our study, DNA methylation had good discrimination even in patients with negative hrHPV results (OR 4.727, *p* = 0.027). The triage capacity of DNA methylation in our study was in accordance with previous reports [28, 35]. However, studies on various DNA methylation triage methods in the Chinese population have reached conflicting conclusions [37–39]. These discrepancies reflect bias associated with the selected study populations and candidate genes.

Based on these findings, we suggest that DNA methylation can serve as an independent screening method for CIN2+ lesions. Despite describing other types of DNA methylation, there are several supporting studies [17, 28, 30, 40–42]. In the study by Boers et al., DNA methylation of a combination of genes (*C13ORF18*/*JAM3/ANKRD18CP*) had higher specificity than hrHPV after a positive Pap smear and had comparable diagnostic accuracies to those of Pap smear in hrHPV-positive scrapings [28]. Their findings together with others suggest that DNA methylation testing may constitute a replacement of HPV DNA testing altogether with only modest improvements in test technology [13].

Methylation assays are relatively easy to set up, perform, and automate. Furthermore, DNA methylation assays exhibit competitive performance with other current triage options at the forefront of reflex triage tests for women who are positive for hrHPV [13]. Testing can even be performed directly from self-collected specimens [14]. In our study, methylation testing achieved a success rate of 98.4% (306/311) when using residual cytology samples stored in the previous year. The good quality of the assay also guarantees the independent utilization of DNA methylation for cervical cancer screening. Regardless, as Lorincz et al. [14] noted in 2014, epigenetic biomarkers need to be considered within the context of differential diagnostic situations and different independent sources, with all the strengths and limitations of the compared tests in full view. Because we achieved favorable results in this exploratory study, we initiated a validation trial among patients with various definitive gynecological pathologies (NCT03960879 study registered at *clinicaltrials.gov*) in the study center to confirm our hypothesis.

We found that the severity of cervical lesions was associated with the proportion of DNA methylation, and these data were significant for CIN3, cervical SCC, endocervical ADC, and even other gynecological carcinomas (Table 2). These findings are consistent with previous reports [31, 43]. The combination of methylation and hrHPV also showed excellent discriminating capacity. However, compared with inflammation or CIN1, a significantly increased proportion of hrHPV positivity was found among CIN3 and SCC cases but with similar ratios among endocervical ADC and other malignancies. *EPB41L3* methylation and HPV types in CIN1 suggest that progression from a normal epithelium to CIN1 or CIN3 is usually promoted by the same HPV type but occurs via distinct DNA epigenotypes [44].

In our study, DNA methylation was better than hrHPV for discriminating cervical ADC. Patients with cervical ADC often present a series of characteristic clinical features, such as a high positive lymph node rate, distant metastasis rate, and recurrence rate, corresponding to a poor prognosis [45, 46]. A much lower prevalence of hrHPV is found in ADC than in squamous carcinomas, 100% of which are positive for HPV infection [47–51]. HPV-negative endocervical ADCs of the usual type vary in frequency from 4.8 to 40.0% across China [50, 52], and early diagnosis of HPV-negative cervical ADCs is still challenging [53]. The favorable discrimination ability of DNA methylation in our study indicates that it is probably an applicable and feasible method for the detection and diagnosis of ADC. Nevertheless, the entire cohort of ADC in our study was too small to achieve conclusions as definite as the situations in CIN2+ and SCC. For other types of ADC, such as ADC in situ or special subtypes (mucinous, endometrioid, etc.), the role of DNA methylation needs to be verified in our validation trial (NCT03960879). SCC and ADC may also have different specific DNA methylation types [54, 55], and these differences deserve further exploration.

In the current study, we determined the cutoff values for *EPB41L3* and *JAM3* methylation for differentiating CIN2+ from other cervical lesions. First, as for all patients with abnormal cytology and/or hrHPV results, the diagnostic accuracies of cytology, hrHPV, and cytology plus hrHPV were limited by selection bias in this cohort, and the accuracies of DNA methylation need to be reliably reproduced. Table 3 just provided an example for this comparison, and a validated result is still expected in an unbiased trial (NCT03960879), which is still going on. Second, DNA methylation testing was not performed for HIV-positive patients or patients with ADC other than the endocervical subtype; regardless, previous studies of immunocompromised populations have shown that DNA methylation has an essential impact on the detection of CIN2+ [56, 57]. Third, all cervical histological pathology results in this study were based on cervical biopsies, which may involve small but maybe essential differences compared to the final pathology results, likely leading to accurate analysis and bias. Last, we did not perform a follow-up of the prognosis of the patients, limiting the interpretation of the DNA methylation data with regard to the carcinogenesis and progression of cervical cancer, as demonstrated in other studies [58]. These limitations may be resolved in future validation trials.

## Conclusions

In this exploratory study, methylation of *EPB41L3* plus *JAM3* had a similar diagnostic accuracy to that of hrHPV for detecting CIN2+. DNA methylation may be an alternative screening method. A further validation trial is needed to confirm these findings.

## Supplementary information

**Additional file 1:.** Supplementary Table 1. Raw data for the study.

## Data Availability

All data for this study are contained in the supplement file.

## Abbreviations

95% CI: 95% confidence interval
ADC: Adenocarcinoma
CIN: Cervical intraepithelial neoplasia
CIN2+: Cervical intraepithelial neoplasia 2 or more severe lesions
HGSC: High-grade serous carcinoma
HSIL: High-grade intraepithelial lesions
hrHPV: High-risk human papillomavirus
LEEP: Loop electrosurgical excision procedure
LSIL: Low-grade intraepithelial lesions
NPV: Negative predictive value
OR: Odds ratio
PCR: Polymerase chain reaction
PPV: Positive predictive value
ROC: Receiver operating characteristic curve
SCC: Squamous cell carcinoma
SPSS: Statistical Product and Service Solutions
